# Crystal structure of a dicationic Pd^II^ dimer containing a 2-[(diisopropylphosphanyl)methyl]quinoline-8-thiolate pincer ligand

**DOI:** 10.1107/S2056989021012561

**Published:** 2022-01-01

**Authors:** Arnaud Clerc, Nathalie Saffon-Merceron, Julien Monot, Blanca Martin Vaca, Didier Bourissou

**Affiliations:** a Laboratoire Hétérochimie Fondamentale et Appliquée, LHFA UMR-CNRS 5069, Université Paul Sabatier, 118 route de Narbonne, 31062 Toulouse Cedex 09, France; b Université de Toulouse III Paul Sabatier, Institut de Chimie de Toulouse, ICT, UAR 2599, 118, route de Narbonne, F-31062 Toulouse, France

**Keywords:** crystal structure, Pd^II^ pincer complex, dimer, S-bridging coordination, quinoline

## Abstract

A dicationic Pd^II^ dimer, bis{2-[(diisopropylphosphanyl)methyl]quinoline-8-thiolato}palladium(II) bis(hexafluoridoantimonate) dichloromethane monosolvate, [Pd_2_(C_32_H_42_N_2_P_2_S_2_)](SbF_6_)_2_·CH_2_Cl_2_, containing a 2-[(diisopropylphos­phanyl)methyl]quinoline-8-thiolate pincer ligand, was isolated and its crystal structure determined. The title compound crystallizes in the ortho­rhom­bic space group *Pbca*. A dimeric structure is formed by bridging coordination of the S atoms. The geometry of the butterfly-shaped Pd_2_S_2_ core is bent, with a hinge angle of 108.0 (1)° and a short Pd⋯Pd distance of 2.8425 (7) Å. These values are the lowest measured compared to ten dicationic dimers with a Pd_2_S_2_ core featuring sulfur atoms embedded in a chelating ligand. One of the two hexa­fluorido­anti­monate anions is disordered over two sets of positions with site-occupancy factors of 0.711 (5) and 0.289 (5). The crystal structure is stabilized by many C—H⋯F and C—H⋯π inter­actions, forming a supra­molecular network.

## Abstract 2

A dicationic Pd^II^ dimer containing a [2-(methyl­enphosphan­yl)-8-thiol­ate­quinoline] pincer ligand was isolated and its crystal structure determined.

## Chemical context

**Figure 1:**
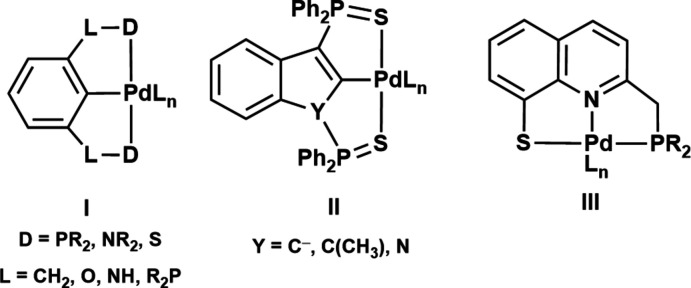
Schematic representation of Pd pincer complexes **I**–**III**

The stereoelectronic properties of transition-metal complexes can be finely modulated thanks to the ligands introduced on the metal coordination sphere, and this plays a fundamental role in organometallic chemistry. Over the past two decades, impressive developments have been achieved with pincer complexes, which nicely illustrate how the properties and reactivity of a complex can be adjusted through ligand modifications (Morales-Morales, 2018 ▸). In pincer complexes, the central *M*—*X* bond is enforced by the coordination of two peripheral donor groups (*D*), and the chelating rigid nature of the monoanionic *DXD* pincer ligand bestows a unique balance between stability and reactivity. This has led to spectacular catalytic developments, including with pincer complexes based on Pd, a transition metal that occupies a central place in organometallic catalysis. As far as Pd is concerned, the main topology of the used monoanionic pincer ligands consists of an aryl central moiety featuring two coordinating side arms, as illustrated in Fig. 1 ▸ (model **I**). These complexes have been successfully applied to C—C or C—*X* bond-forming catalytic transformations. The impact of the side groups (coordinating atom and linker) on the catalytic performances has been explored (Selander *et al.*, 2011 ▸). We have developed new models of Pd pincer complexes varying the aromatic central ring, introducing indenyl and indolyl moieties (model **II** in Fig. 1 ▸). The nature of the central ring was found to significantly impact the catalytic activity of the Pd complexes in the allyl­ation of amines (Lisena *et al.*, 2013 ▸).

Seeking to further modify the structure of the Pd pincer complexes so that the catalytic activity can be modulated, we now aim to incorporate an extended π-system as the central moiety (so that rigidity is increased). We have thus designed and prepared a pincer PNS Pd complex based on a 8-thiol­ate-quinoline featuring a methyl­enephosphine side arm (model **III** in Fig. 1 ▸). We report herein that when cationizing the corresponding chloro palladium pincer complex **1** with AgSbF_6_, a dimeric dicationic species **2** crystallized with a tight *S*-bridging assembling of the two quinoline-based PNS Pd pincer fragments. The structural features are discussed. It is worth noting that we have previously reported S-bridged homo and hetero polymetallic species derived from Pd pincer complexes of type **II** (Nebra *et al.*, 2011 ▸, 2012 ▸). 
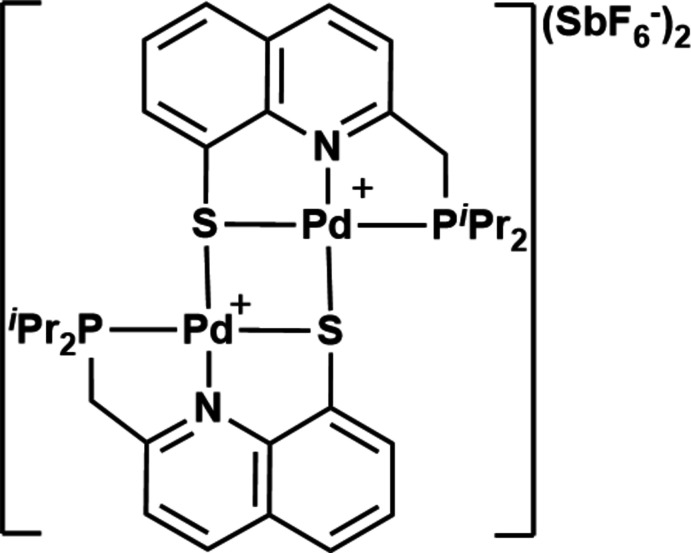


## Structural commentary

**Figure 2:**
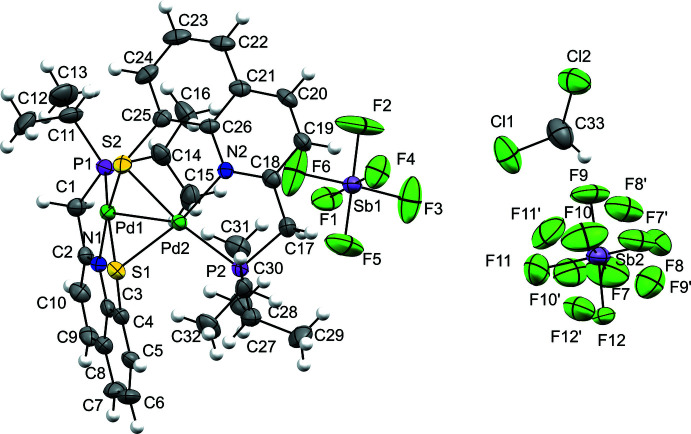
The mol­ecular structure of the title compound with the atom numbering. Displacement ellipsoids are drawn at the 50% probability level.

X-ray diffraction of the yellow crystals obtained from **2(SbF_6_)_2_** revealed a dimeric structure, composed of two cationic PNSPd fragments, that crystallizes in the ortho­rhom­bic system and *Pbca* space group (Figs. 2 ▸ and 3 ▸; selected bond lengths and bond angles are given in Table 1 ▸). The dicationic nature of the structure is confirmed by the presence of two SbF_6_^−^ units per dimer. The two PNSPd fragments are connected to each other by two bridging S atoms. The S donor atom of each PNSPd fragment completes the coordination sphere of the other, forming a Pd_2_S_2_ diamond core.

For each PNSPd fragment, besides the two bridging S atoms, the Pd atom is coordinated by one N atom and one P atom, completing a tetra­coordinate sphere that deviates slightly from square-planar geometry (deviation estimated by the τ index, with values of 0.15 and 0.16 for Pd1 and Pd2, respectively) (Yang *et al.*, 2007 ▸). The Pd—N and the Pd—P bond lengths are almost identical for the two fragments [Pd1—N1 = 2.027 (5), Pd2—N2 = 2.027 (5) Å and Pd1—P1 = 2.2455 (18), Pd2—P2 = 2.2417 (18) Å], and the values are in the range of those observed for quinoline/phosphine chelate Pd complexes (Mori *et al.*, 2021 ▸; Scharf *et al.*, 2014 ▸ for example). The coordination environment around each Pd atom and the quinoline moiety is approximately planar [dihedral angles of 13.1 (1)° for Pd1 and 2.3 (1)° for Pd2, as estimated by the dihedral angle between the mean planes of the two fragments].

As for the Pd_2_S_2_ core, the two Pd—S bond lengths for each Pd atom are slightly different and, inter­estingly, the bonds between the Pd atoms and the bridging S atom of the other fragment are shorter [2.3149 (16) and 2.3184 (16) for Pd1—S2 and Pd2—S1, respectively] than the bonds between the Pd atoms and the chelating S atom of the pincer ligand [2.3657 (17) and 2.3602 (17) for Pd1—S1 and Pd2—S2, respectively]. This is most likely due to the rigidity of the 8-thio-quinoline moiety (the C3—C4—S1 and C26—C27—S2 angles deviate from 120° by less than 2°). The two S atoms are noticeably pyramidalized (ΣS = 287 and 290° for S1 and S2, respectively). The hinge angle of the core unit (involving the two [S,Pd,S] planes) has a value of 108.0 (1)°, which is in fact the lowest value reported for such kind of dicationic species with a Pd_2_S_2_ core (see the *Database survey* section). This results in a rather short Pd1—Pd2 distance of 2.8425 (7) Å, which is significantly shorter than the sum of van der Waals radii (4.10 Å; Batsanov *et al.*, 2001 ▸) and exceeds the sum of the covalent radii (2.78 Å; Cordero *et al.*, 2008 ▸) by only 2%.

## Supra­molecular features

**Figure 4:**
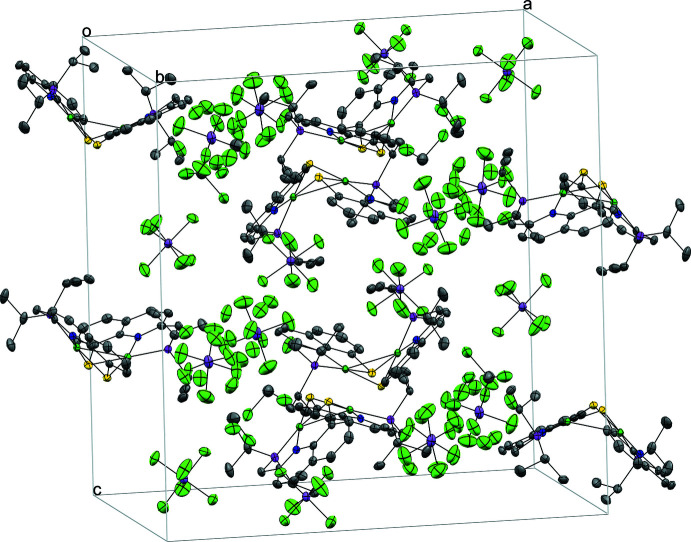
A partial packing diagram of **2(SBF_6_)_2_**; H atoms, and solvent omitted for clarity.

**Figure 5:**
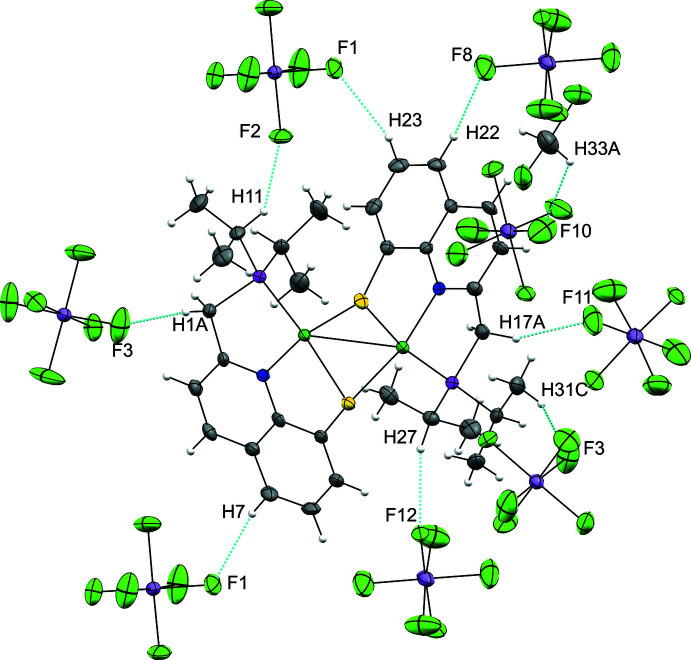
C—H⋯F hydrogen bonds (blue dotted lines).

The crystal packing of the title compound, illustrated in Fig. 4 ▸, involves weak intra­molecular C—H⋯*Cg* contacts, and inter­molecular C—H⋯F contacts between the cations and anions, which link the components in a three-dimensional network (Table 2 ▸, Figs. 5 ▸ and 6 ▸). No classical hydrogen-bonding inter­actions were found.

**Figure 6:**
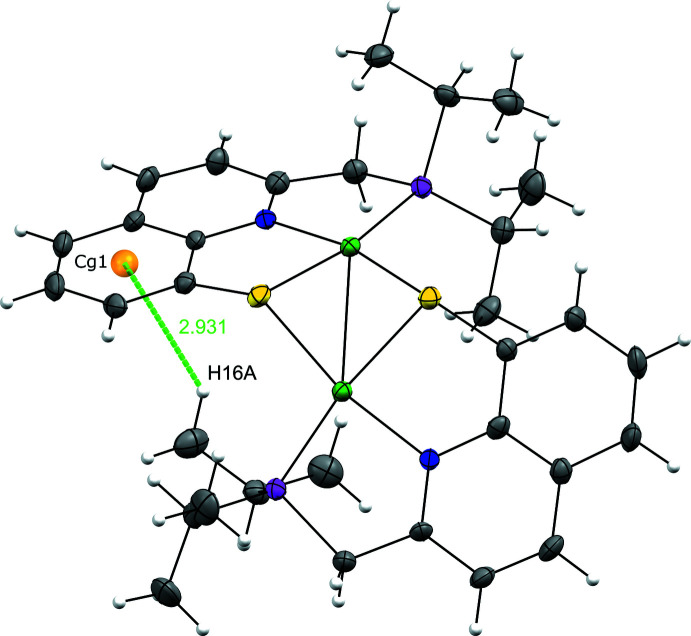
C—H⋯*Cg* contact.

Each dicationic unit is surrounded by eight SbF_6_^−^ anions, engaged in weak C—H⋯F contacts with C⋯F distances in the range 3.128 (9)–3.172 (13) Å (associated with H⋯F distances in the range 2.27–2.54 Å) (Fig. 5 ▸). As for the SbF_6_^−^ anions, two different situations can be observed. One of the anions (containing Sb1) displays weak C—H⋯F contacts with C—H bonds from five different dicationic units, while the other one (containing Sb2), inter­acts weakly with C—H bonds from three dicationic units and from a CH_2_Cl_2_ solvent mol­ecule. Finally, an intra­molecular C—H⋯*Cg* short contact is observed between one of the CH_3_ of the ^*i*^Pr groups of one PNSPd pincer fragment (Pd2) and the benzo ring of the quinoline moiety of the other fragment [C16⋯*Cg*1 = 3.701 (8) Å, associated with a H16*A*⋯*Cg*1 distance of 2.93 Å] (Fig. 6 ▸). It should be noted that a significantly longer distance (H28*B*⋯*Cg*2 of 3.2 Å) is observed for the other part of the unit (CH_3_ group of the Pd2 fragment with the benzo ring of the other), indicating a non-symmetrical organization of the dimer.

## Database survey

To the best of our knowledge, structures of quinoline-based PNSPd dicationic dimers as described herein have not been reported previously. A structure survey was carried out in the Cambridge Structural Database (CSD version 5.42, update of November 2020; Groom *et al.*, 2016 ▸). It revealed 28 hits for dicationic dimers with a Pd_2_S_2_ core, of which ten can be compared with the title compound as they feature the sulfur atoms embedded in a chelating ligand [refcodes CUYLIT (Kouno *et al.*, 2015 ▸), NORGEG (Albinati *et al.*, 1997 ▸), NOXVAZ (Chen *et al.* 2015 ▸), POTMUG (Kersting, 1998 ▸), QOCCUG (Su *et al.*, 2000 ▸), SELGUL (Leung *et al.*, 1998 ▸), TEGWUY (Cabeza *et al.*, 2006 ▸), TIXLOE (Mane *et al.*, 2019 ▸), XAHBUI (Nayan Sharma *et al.*, 2015 ▸), XULYUZ (Azizpoor Fard *et al.*, 2015 ▸)]. Hinge angles in the range 115.3–156.6° were measured for these compounds, all values higher than that measured for the title compound [108.0 (1)°].

## Synthesis and crystallization

A solution of PNS-Pd-Cl **1** (Scharf *et al.*, 2014 ▸) (1.0 equiv., 0.1 *M*) was added dropwise over 5 min to a suspension of AgSbF_6_ (1.0 equiv.) in CH_2_Cl_2_ (0.1 *M*) at 195 K. After the addition, the reaction mixture was allowed to quickly warm up to room temperature and was stirred for 2 h. The reaction was then filtered *via* canula, and the solvent was removed *in vacuo* to yield the corresponding dicationic complex as a reddish powder (95%). X-ray quality crystals were grown by slow diffusion at 273 K of pentane into a concentrated solution of **2** in CH_2_Cl_2_. ^1^H NMR (300 MHz, CD_2_Cl_2_): δ = 8.60 (*d*, *J =* 8.5 Hz, 2H), 8.23 (*dd*, *J =* 7.5, 1.2 Hz, 2H), 8.13 (*dd*, *J =* 8.5, 1.2 Hz, 2H), 7.87–7.75 (*m*, 4H), 4.16 (*dd*, *J =* 18.9, 9.7 Hz, 2H), 3.86 (*dd*, *J =* 18.9, 11.2 Hz, 2H), 2.47 (*m*, 2H), 1.79 (*dd*, *J =* 20.1, 7.1 Hz, 6H), 1.49 (*dd*, *J =* 17.4, 6.9 Hz, 6H), 1.28 (*m*, 2H), 0.82 (*dd*, *J =* 16.1, 6.9 Hz, 6H), 0.08 (*dd*, *J =* 19.7, 7.1 Hz, 6H).

## Refinement

Crystal data, data collection and structure refinement details are summarized in Table 3 ▸. One of the two hexa­fluorido­anti­monate anions is disordered over two positions, for which occupancies were refined, converging to 0.711 (5) and 0.289 (5). SAME, DELU and SIMU restraints were applied (Sheldrick, 2015*b* ▸). All H atoms were fixed geometrically and treated as riding with C—H = 0.95 Å (aromatic), 0.98 Å (CH_3_), 0.99 Å (CH_2_) or 1.0 Å (CH), with *U*_iso_(H) = 1.2*U*_eq_(CH, CH_2_) or *1.5U*_eq_(CH_3_)*.*

## Supplementary Material

Crystal structure: contains datablock(s) I. DOI: 10.1107/S2056989021012561/zq2270sup1.cif

Structure factors: contains datablock(s) I. DOI: 10.1107/S2056989021012561/zq2270Isup3.hkl

CCDC reference: 2124311

Additional supporting information:  crystallographic information; 3D view; checkCIF report

## Figures and Tables

**Figure 3:**
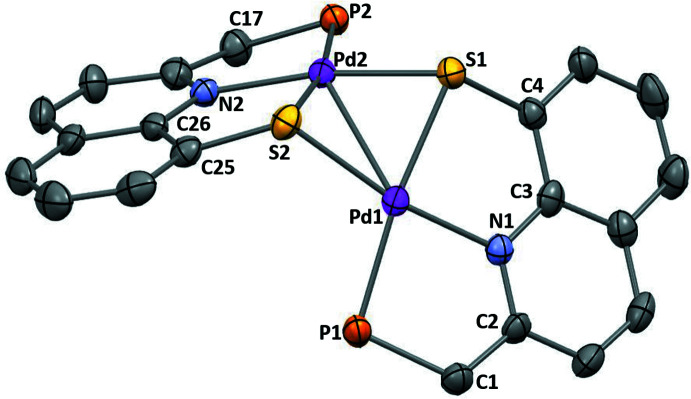
Detail of the mol­ecular structure of **2^2+^**, showing the main atom-numbering scheme and displacement ellipsoids at the 50% probability level. H atoms and ^*i*^Pr groups have been omitted for clarity.

**Table 1:** Selected geometric parameters (Å, °)

Pd1—N1	2.027 (5)	Pd2—S1	2.3184 (16)
Pd1—P1	2.2455 (18)	Pd2—S2	2.3602 (17)
Pd1—S2	2.3149 (16)	P1—C1	1.825 (6)
Pd1—S1	2.3657 (17)	P2—C17	1.836 (6)
Pd1—Pd2	2.8425 (7)	S1—C4	1.784 (6)
Pd2—N2	2.027 (5)	S2—C25	1.774 (7)
Pd2—P2	2.2417 (18)		
			
N1—Pd1—P1	83.86 (15)	N2—Pd2—P2	85.21 (15)
N1—Pd1—S2	168.93 (15)	N2—Pd2—S1	167.55 (15)
P1—Pd1—S2	106.75 (6)	P2—Pd2—S1	106.34 (6)
N1—Pd1—S1	86.49 (15)	N2—Pd2—S2	86.13 (15)
P1—Pd1—S1	169.03 (6)	P2—Pd2—S2	170.20 (6)
S2—Pd1—S1	82.64 (6)	S1—Pd2—S2	82.69 (6)
N1—Pd1—Pd2	117.54 (14)	N2—Pd2—Pd1	114.86 (14)
P1—Pd1—Pd2	129.40 (5)	P2—Pd2—Pd1	136.83 (5)
S2—Pd1—Pd2	53.28 (4)	S1—Pd2—Pd1	53.40 (4)
S1—Pd1—Pd2	51.89 (4)	S2—Pd2—Pd1	51.83 (4)
Pd2—S1—Pd1	74.71 (5)	Pd1—S2—Pd2	74.88 (5)

**Table 2:** Hydrogen-bond geometry (Å, °) *Cg*1 is the centroid of the C21–C26 ring.

*D*—H⋯*A*	*D*—H	H⋯*A*	*D*⋯*A*	*D*—H⋯*A*
C1—H1*A*⋯F3^i^	0.99	2.34	3.305 (9)	166
C7—H7⋯F1^ii^	0.95	2.37	3.229 (8)	151
C11—H11⋯F2^iii^	1.00	2.27	3.128 (9)	143
C17—H17*A*⋯F11^iv^	0.99	2.41	3.279 (10)	147
C22—H22⋯F8^v^	0.95	2.33	3.190 (11)	150
C23—H23⋯F1^iii^	0.95	2.53	3.396 (9)	152
C27—H27⋯F12^vi^	1.00	2.43	3.322 (10)	148
C31—H31*C*⋯F3^iv^	0.98	2.50	3.399 (9)	152
C33—H33*A*⋯F10	0.99	2.54	3.172 (13)	122
C16—H16*A*⋯*Cg*1	0.98	2.93	3.701 (8)	136

Symmetry codes: (i) x+{\script{1\over 2}}, -y+{\script{3\over 2}}, -z+1; (ii) -x+1, -y+2, -z+1; (iii) -x+1, -y+1, -z+1; (iv) x, -y+{\script{3\over 2}}, z+{\script{1\over 2}}; (v) -x+{\script{1\over 2}}, -y+1, z+{\script{1\over 2}}; (vi) -x+{\script{1\over 2}}, -y+2, z+{\script{1\over 2}}.

**Table 3:** Experimental details

Crystal data	
Chemical formula	[Pd_2_(C_32_H_42_N_2_P_2_S_2_)](SbF_6_)_2_·CH_2_Cl_2_
*M*_r_	1349.96
Crystal system, space group	Orthorhombic, *P* *b* *c* *a*
Temperature (K)	193
*a*, *b*, *c* (Å)	23.5167 (19), 16.1492 (14), 24.0414 (18)
*V* (Å^3^)	9130.3 (13)
*Z*	8
Radiation type	Mo *K*α
μ (mm^−1^)	2.30
Crystal size (mm)	0.10 × 0.08 × 0.04

Data collection	
Diffractometer	Bruker Kappa APEXII CCD Quazar
Absorption correction	Multi-scan (*SADABS*; Bruker, 2014 ▸)
*T*_min_, *T*_max_	0.677, 0.728
No. of measured, independent and observed [*I* > 2σ(*I*)] reflections	152552, 9812, 6263
*R*_int_	0.122
(sin θ/λ)_max_ (Å^−1^)	0.637

Refinement	
*R*[*F* ^2^ > 2σ(*F* ^2^)], *wR*(*F* ^2^), *S*	0.046, 0.113, 1.01
No. of reflections	9812
No. of parameters	577
No. of restraints	213
H-atom treatment	H-atom parameters constrained
Δρ_max_, Δρ_min_ (e Å^−3^)	1.50, −1.07

Computer programs: *APEX2* (Bruker, 2014 ▸) and *SHELXT* (Sheldrick, 2015*a* ▸), *SHELXL2018/3* (Sheldrick, 2015*b* ▸), *SHELXTL* (Sheldrick, 2008 ▸) and *Mercury* (Macrae *et al.*, 2020 ▸), *PLATON* (Spek, 2020 ▸) and *publCIF* (Westrip, 2010 ▸).

## supplementary crystallographic information

### Crystal data

**Table:**

[Pd_2_(C_32_H_42_N_2_P_2_S_2_)](SbF_6_)_2_·CH_2_Cl_2_	*D*_x_ = 1.964 Mg m^−^^3^
*M**_r_* = 1349.96	Mo *K*α radiation, λ = 0.71073 Å
Orthorhombic, *P**b**c**a*	Cell parameters from 9991 reflections
*a* = 23.5167 (19) Å	θ = 3.0–22.0°
*b* = 16.1492 (14) Å	µ = 2.30 mm^−^^1^
*c* = 24.0414 (18) Å	*T* = 193 K
*V* = 9130.3 (13) Å^3^	Plate, yellow
*Z* = 8	0.10 × 0.08 × 0.04 mm
*F*(000) = 5232	

### Data collection

**Table:**

Bruker Kappa APEXII CCD Quazar diffractometer	6263 reflections with *I* > 2σ(*I*)
Radiation source: Incoatec microfocus sealed tube	*R*_int_ = 0.122
Phi and ω scans	θ_max_ = 26.9°, θ_min_ = 1.9°
Absorption correction: multi-scan (SADABS; Bruker, 2014)	*h* = −29→29
*T*_min_ = 0.677, *T*_max_ = 0.728	*k* = −20→20
152552 measured reflections	*l* = −30→30
9812 independent reflections	

### Refinement

**Table:**

Refinement on *F*^2^	Primary atom site location: dual
Least-squares matrix: full	Secondary atom site location: difference Fourier map
*R*[*F*^2^ > 2σ(*F*^2^)] = 0.046	Hydrogen site location: inferred from neighbouring sites
*wR*(*F*^2^) = 0.113	H-atom parameters constrained
*S* = 1.01	*w* = 1/[σ^2^(*F*_o_^2^) + (0.0384*P*)^2^ + 45.7164*P*] where *P* = (*F*_o_^2^ + 2*F*_c_^2^)/3
9812 reflections	(Δ/σ)_max_ = 0.002
577 parameters	Δρ_max_ = 1.50 e Å^−^^3^
213 restraints	Δρ_min_ = −1.07 e Å^−^^3^

### Special details

**Table:**

Geometry. All esds (except the esd in the dihedral angle between two l.s. planes) are estimated using the full covariance matrix. The cell esds are taken into account individually in the estimation of esds in distances, angles and torsion angles; correlations between esds in cell parameters are only used when they are defined by crystal symmetry. An approximate (isotropic) treatment of cell esds is used for estimating esds involving l.s. planes.

### Fractional atomic coordinates and isotropic or equivalent isotropic displacement parameters (Å^2^)

**Table:**

	*x*	*y*	*z*	*U*_iso_*/*U*_eq_	Occ. (<1)
Pd1	0.57179 (2)	0.75620 (3)	0.64839 (2)	0.02433 (12)	
Pd2	0.45502 (2)	0.74339 (3)	0.67769 (2)	0.02348 (12)	
P1	0.61946 (7)	0.69467 (10)	0.57882 (7)	0.0291 (4)	
P2	0.37442 (7)	0.81155 (10)	0.65817 (7)	0.0268 (4)	
S1	0.51907 (7)	0.84184 (10)	0.70934 (6)	0.0284 (4)	
S2	0.52977 (7)	0.65162 (10)	0.69984 (7)	0.0304 (4)	
N1	0.5978 (2)	0.8622 (3)	0.6110 (2)	0.0248 (11)	
N2	0.4144 (2)	0.6430 (3)	0.6462 (2)	0.0243 (11)	
C1	0.6648 (3)	0.7812 (4)	0.5584 (3)	0.0311 (15)	
H1A	0.701799	0.776732	0.577766	0.037*	
H1B	0.671982	0.778550	0.517885	0.037*	
C2	0.6380 (3)	0.8623 (4)	0.5723 (3)	0.0270 (14)	
C3	0.5708 (3)	0.9343 (4)	0.6262 (3)	0.0285 (14)	
C4	0.5282 (3)	0.9334 (4)	0.6688 (2)	0.0274 (14)	
C5	0.5009 (3)	1.0053 (4)	0.6821 (3)	0.0355 (16)	
H5	0.472562	1.005157	0.710333	0.043*	
C6	0.5141 (3)	1.0796 (4)	0.6546 (3)	0.046 (2)	
H6	0.493775	1.128646	0.663638	0.056*	
C7	0.5560 (3)	1.0825 (4)	0.6149 (3)	0.0418 (18)	
H7	0.565065	1.133373	0.597251	0.050*	
C8	0.5854 (3)	1.0096 (4)	0.6005 (3)	0.0328 (16)	
C9	0.6293 (3)	1.0089 (4)	0.5603 (3)	0.0352 (16)	
H9	0.640761	1.058936	0.542901	0.042*	
C10	0.6549 (3)	0.9363 (4)	0.5467 (3)	0.0360 (16)	
H10	0.684390	0.935601	0.519667	0.043*	
C11	0.6661 (3)	0.6081 (4)	0.5938 (3)	0.0432 (19)	
H11	0.641178	0.558737	0.599777	0.052*	
C12	0.6989 (4)	0.6226 (5)	0.6488 (3)	0.059 (2)	
H12A	0.722728	0.574233	0.656806	0.089*	
H12B	0.671824	0.630923	0.679202	0.089*	
H12C	0.723057	0.671770	0.645046	0.089*	
C13	0.7053 (4)	0.5874 (5)	0.5455 (4)	0.067 (3)	
H13A	0.731357	0.633794	0.538993	0.101*	
H13B	0.682578	0.577628	0.511957	0.101*	
H13C	0.727221	0.537571	0.554384	0.101*	
C14	0.5769 (3)	0.6701 (4)	0.5169 (3)	0.0381 (17)	
H14	0.603766	0.662319	0.485083	0.046*	
C15	0.5379 (3)	0.7421 (5)	0.5027 (3)	0.055 (2)	
H15A	0.515430	0.728286	0.469620	0.082*	
H15B	0.560770	0.791607	0.495211	0.082*	
H15C	0.512291	0.752911	0.534025	0.082*	
C16	0.5430 (4)	0.5895 (5)	0.5238 (3)	0.055 (2)	
H16A	0.515351	0.596008	0.553935	0.083*	
H16B	0.569058	0.544039	0.532664	0.083*	
H16C	0.522942	0.577044	0.489023	0.083*	
C17	0.3362 (3)	0.7327 (4)	0.6178 (3)	0.0331 (16)	
H17A	0.295987	0.731432	0.629853	0.040*	
H17B	0.336994	0.748252	0.577947	0.040*	
C18	0.3612 (3)	0.6488 (4)	0.6244 (3)	0.0311 (15)	
C19	0.3333 (3)	0.5775 (4)	0.6056 (3)	0.0341 (16)	
H19	0.295630	0.581288	0.591709	0.041*	
C20	0.3598 (3)	0.5032 (4)	0.6072 (3)	0.0351 (17)	
H20	0.340085	0.455065	0.595184	0.042*	
C21	0.4163 (3)	0.4960 (4)	0.6265 (3)	0.0308 (15)	
C22	0.4467 (3)	0.4207 (4)	0.6265 (3)	0.0385 (18)	
H22	0.428883	0.371171	0.614199	0.046*	
C23	0.5014 (4)	0.4194 (4)	0.6440 (3)	0.0446 (19)	
H23	0.522041	0.368879	0.643264	0.053*	
C24	0.5279 (3)	0.4912 (4)	0.6632 (3)	0.0383 (17)	
H24	0.566472	0.488814	0.675010	0.046*	
C25	0.4995 (3)	0.5650 (4)	0.6654 (2)	0.0283 (15)	
C26	0.4427 (3)	0.5686 (4)	0.6464 (2)	0.0257 (14)	
C27	0.3749 (3)	0.9063 (4)	0.6176 (3)	0.0360 (16)	
H27	0.388653	0.951774	0.642469	0.043*	
C28	0.4167 (3)	0.8992 (5)	0.5693 (3)	0.0457 (19)	
H28A	0.402839	0.857879	0.542684	0.069*	
H28B	0.454011	0.882220	0.583404	0.069*	
H28C	0.420108	0.952991	0.550674	0.069*	
C29	0.3144 (3)	0.9306 (5)	0.5971 (4)	0.058 (2)	
H29A	0.315917	0.984838	0.578951	0.086*	
H29B	0.288394	0.933056	0.628914	0.086*	
H29C	0.300799	0.889035	0.570524	0.086*	
C30	0.3325 (3)	0.8286 (4)	0.7209 (3)	0.0330 (16)	
H30	0.293211	0.845049	0.709329	0.040*	
C31	0.3281 (3)	0.7487 (5)	0.7547 (3)	0.0458 (19)	
H31A	0.366266	0.730653	0.765698	0.069*	
H31B	0.310159	0.705558	0.732012	0.069*	
H31C	0.305098	0.758646	0.788001	0.069*	
C32	0.3579 (3)	0.8998 (5)	0.7557 (3)	0.051 (2)	
H32A	0.337543	0.903830	0.791199	0.077*	
H32B	0.354032	0.951989	0.735303	0.077*	
H32C	0.398210	0.888704	0.762806	0.077*	
Sb1	0.34706 (2)	0.68208 (3)	0.44208 (2)	0.03304 (12)	
F1	0.4044 (2)	0.7271 (3)	0.3981 (2)	0.0681 (14)	
F2	0.3620 (3)	0.5810 (3)	0.4107 (3)	0.119 (3)	
F3	0.2940 (2)	0.7045 (5)	0.38843 (19)	0.099 (2)	
F4	0.28965 (19)	0.6387 (3)	0.48634 (19)	0.0688 (14)	
F5	0.3330 (3)	0.7845 (3)	0.4738 (3)	0.095 (2)	
F6	0.3986 (2)	0.6626 (5)	0.4985 (2)	0.115 (3)	
Sb2	0.15370 (2)	0.80751 (3)	0.14915 (2)	0.04860 (16)	
F7	0.1744 (6)	0.8508 (6)	0.0840 (4)	0.116 (3)	0.711 (5)
F8	0.0865 (4)	0.7708 (5)	0.1235 (5)	0.113 (3)	0.711 (5)
F9	0.1845 (4)	0.7047 (4)	0.1293 (4)	0.089 (3)	0.711 (5)
F10	0.1376 (5)	0.7592 (5)	0.2189 (4)	0.115 (3)	0.711 (5)
F11	0.2224 (3)	0.8451 (5)	0.1768 (4)	0.099 (3)	0.711 (5)
F12	0.1235 (3)	0.9068 (4)	0.1753 (3)	0.0580 (19)	0.711 (5)
F7'	0.0894 (7)	0.7609 (10)	0.1732 (9)	0.082 (4)	0.289 (5)
F8'	0.1563 (10)	0.7305 (11)	0.0906 (8)	0.095 (4)	0.289 (5)
F9'	0.1123 (9)	0.8545 (12)	0.0867 (7)	0.098 (4)	0.289 (5)
F10'	0.2132 (8)	0.8578 (12)	0.1071 (10)	0.094 (4)	0.289 (5)
F11'	0.1990 (9)	0.7690 (13)	0.1991 (8)	0.114 (5)	0.289 (5)
F12'	0.1486 (10)	0.8969 (11)	0.1936 (9)	0.091 (4)	0.289 (5)
C33	0.1687 (4)	0.5737 (6)	0.2499 (4)	0.072 (3)	
H33A	0.133730	0.607493	0.246158	0.087*	
H33B	0.190008	0.577838	0.214525	0.087*	
Cl1	0.21006 (11)	0.61408 (19)	0.30332 (10)	0.0835 (9)	
Cl2	0.14970 (13)	0.47124 (16)	0.26099 (10)	0.0816 (8)	

### Atomic displacement parameters (Å^2^)

**Table:**

	*U*^11^	*U*^22^	*U*^33^	*U*^12^	*U*^13^	*U*^23^
Pd1	0.0225 (2)	0.0220 (2)	0.0285 (2)	−0.0019 (2)	−0.0001 (2)	0.0012 (2)
Pd2	0.0227 (2)	0.0216 (2)	0.0262 (2)	−0.0024 (2)	0.0003 (2)	−0.0004 (2)
P1	0.0255 (9)	0.0231 (9)	0.0385 (9)	−0.0015 (7)	0.0033 (8)	−0.0045 (7)
P2	0.0244 (9)	0.0243 (8)	0.0318 (9)	−0.0001 (7)	0.0001 (7)	−0.0035 (7)
S1	0.0293 (9)	0.0279 (9)	0.0279 (8)	−0.0046 (7)	0.0004 (7)	−0.0035 (7)
S2	0.0302 (9)	0.0280 (9)	0.0329 (8)	−0.0021 (7)	−0.0019 (7)	0.0081 (7)
N1	0.024 (3)	0.021 (3)	0.029 (3)	0.000 (2)	−0.001 (2)	0.000 (2)
N2	0.021 (3)	0.024 (3)	0.028 (3)	−0.001 (2)	0.001 (2)	−0.001 (2)
C1	0.027 (4)	0.029 (3)	0.038 (4)	0.000 (3)	0.006 (3)	0.000 (3)
C2	0.019 (3)	0.028 (3)	0.034 (3)	−0.005 (3)	0.002 (3)	0.000 (3)
C3	0.023 (3)	0.029 (4)	0.034 (3)	−0.007 (3)	−0.007 (3)	0.001 (3)
C4	0.025 (4)	0.026 (3)	0.031 (3)	−0.002 (3)	−0.003 (3)	−0.005 (3)
C5	0.032 (4)	0.028 (4)	0.047 (4)	−0.005 (3)	0.002 (3)	−0.010 (3)
C6	0.047 (5)	0.022 (4)	0.070 (5)	0.007 (3)	−0.005 (4)	−0.010 (4)
C7	0.041 (5)	0.022 (4)	0.062 (5)	0.002 (3)	0.000 (4)	0.009 (3)
C8	0.031 (4)	0.027 (4)	0.041 (4)	−0.002 (3)	−0.005 (3)	−0.004 (3)
C9	0.036 (4)	0.030 (4)	0.039 (4)	−0.012 (3)	−0.004 (3)	0.011 (3)
C10	0.037 (4)	0.034 (4)	0.037 (4)	−0.010 (3)	0.007 (3)	0.005 (3)
C11	0.035 (4)	0.024 (4)	0.071 (5)	0.007 (3)	0.012 (4)	0.001 (3)
C12	0.047 (5)	0.054 (5)	0.077 (6)	0.018 (4)	−0.015 (5)	0.015 (5)
C13	0.045 (5)	0.054 (5)	0.103 (7)	0.015 (4)	0.021 (5)	−0.009 (5)
C14	0.036 (4)	0.043 (4)	0.036 (4)	−0.005 (4)	0.003 (3)	−0.011 (3)
C15	0.048 (5)	0.070 (6)	0.045 (4)	−0.001 (5)	−0.013 (4)	−0.009 (4)
C16	0.056 (5)	0.065 (6)	0.044 (4)	−0.026 (5)	−0.001 (4)	−0.013 (4)
C17	0.028 (4)	0.034 (4)	0.038 (4)	−0.002 (3)	−0.005 (3)	−0.003 (3)
C18	0.036 (4)	0.031 (4)	0.027 (3)	−0.004 (3)	−0.001 (3)	0.000 (3)
C19	0.035 (4)	0.030 (4)	0.037 (4)	−0.007 (3)	−0.006 (3)	−0.005 (3)
C20	0.043 (4)	0.026 (4)	0.037 (4)	−0.014 (3)	0.000 (3)	−0.004 (3)
C21	0.034 (4)	0.023 (3)	0.035 (4)	−0.004 (3)	0.009 (3)	0.005 (3)
C22	0.055 (5)	0.020 (4)	0.041 (4)	0.000 (3)	0.006 (4)	0.003 (3)
C23	0.063 (6)	0.024 (4)	0.047 (4)	0.011 (4)	0.009 (4)	0.008 (3)
C24	0.039 (4)	0.035 (4)	0.040 (4)	0.012 (3)	0.007 (3)	0.014 (3)
C25	0.030 (4)	0.027 (3)	0.028 (3)	0.000 (3)	0.001 (3)	0.006 (3)
C26	0.031 (4)	0.020 (3)	0.026 (3)	0.001 (3)	0.007 (3)	0.003 (3)
C27	0.040 (4)	0.029 (4)	0.038 (4)	0.000 (3)	−0.003 (3)	0.001 (3)
C28	0.056 (5)	0.043 (4)	0.038 (4)	−0.010 (4)	−0.008 (4)	0.011 (3)
C29	0.055 (5)	0.045 (5)	0.073 (6)	0.010 (4)	−0.014 (5)	0.016 (4)
C30	0.022 (3)	0.039 (4)	0.038 (4)	0.001 (3)	0.002 (3)	−0.006 (3)
C31	0.049 (5)	0.047 (4)	0.042 (4)	−0.009 (4)	0.015 (4)	0.000 (4)
C32	0.049 (5)	0.056 (5)	0.049 (5)	−0.009 (4)	0.010 (4)	−0.023 (4)
Sb1	0.0302 (2)	0.0269 (2)	0.0420 (3)	0.0011 (2)	0.0001 (2)	−0.0041 (2)
F1	0.057 (3)	0.047 (3)	0.100 (4)	−0.007 (2)	0.027 (3)	0.015 (3)
F2	0.129 (6)	0.031 (3)	0.196 (7)	−0.014 (3)	0.092 (5)	−0.030 (4)
F3	0.061 (3)	0.196 (7)	0.040 (3)	0.004 (4)	−0.009 (3)	0.014 (3)
F4	0.046 (3)	0.097 (4)	0.063 (3)	0.002 (3)	0.011 (2)	0.018 (3)
F5	0.121 (5)	0.051 (3)	0.114 (5)	−0.002 (3)	0.023 (4)	−0.037 (3)
F6	0.054 (4)	0.205 (8)	0.087 (4)	0.011 (4)	−0.014 (3)	0.050 (5)
Sb2	0.0383 (3)	0.0289 (3)	0.0786 (4)	−0.0006 (2)	0.0062 (3)	−0.0009 (3)
F7	0.183 (8)	0.084 (5)	0.081 (5)	−0.011 (6)	0.015 (5)	0.008 (4)
F8	0.085 (5)	0.063 (5)	0.191 (7)	−0.004 (4)	−0.046 (5)	−0.031 (5)
F9	0.081 (5)	0.038 (4)	0.149 (7)	0.014 (4)	0.048 (5)	−0.010 (4)
F10	0.153 (7)	0.080 (5)	0.113 (5)	0.024 (5)	0.052 (5)	0.038 (4)
F11	0.048 (4)	0.097 (6)	0.153 (6)	0.004 (4)	−0.011 (4)	−0.032 (5)
F12	0.041 (4)	0.028 (3)	0.105 (5)	0.007 (3)	−0.011 (4)	−0.004 (3)
F7'	0.077 (7)	0.042 (7)	0.126 (9)	−0.007 (6)	0.047 (7)	−0.014 (8)
F8'	0.114 (9)	0.061 (7)	0.110 (8)	−0.003 (7)	0.027 (7)	−0.020 (6)
F9'	0.104 (9)	0.094 (9)	0.095 (8)	0.013 (8)	−0.024 (7)	0.010 (7)
F10'	0.066 (7)	0.068 (8)	0.146 (10)	−0.008 (7)	0.030 (7)	0.015 (8)
F11'	0.109 (9)	0.103 (9)	0.130 (8)	0.039 (8)	−0.028 (8)	0.030 (8)
F12'	0.095 (10)	0.058 (7)	0.120 (9)	−0.001 (7)	−0.005 (8)	−0.028 (7)
C33	0.074 (7)	0.075 (7)	0.068 (6)	−0.026 (6)	−0.003 (5)	0.005 (5)
Cl1	0.0607 (15)	0.122 (2)	0.0683 (15)	−0.0330 (16)	0.0194 (12)	−0.0330 (15)
Cl2	0.115 (2)	0.0626 (15)	0.0675 (15)	−0.0009 (15)	−0.0043 (15)	−0.0104 (12)

### Geometric parameters (Å, º)

**Table:**

Pd1—N1	2.027 (5)	C17—H17A	0.9900
Pd1—P1	2.2455 (18)	C17—H17B	0.9900
Pd1—S2	2.3149 (16)	C18—C19	1.401 (9)
Pd1—S1	2.3657 (17)	C19—C20	1.352 (9)
Pd1—Pd2	2.8425 (7)	C19—H19	0.9500
Pd2—N2	2.027 (5)	C20—C21	1.411 (9)
Pd2—P2	2.2417 (18)	C20—H20	0.9500
Pd2—S1	2.3184 (16)	C21—C26	1.410 (9)
Pd2—S2	2.3602 (17)	C21—C22	1.411 (9)
P1—C11	1.812 (7)	C22—C23	1.355 (10)
P1—C1	1.825 (6)	C22—H22	0.9500
P1—C14	1.836 (7)	C23—C24	1.395 (10)
P2—C27	1.814 (7)	C23—H23	0.9500
P2—C30	1.822 (6)	C24—C25	1.366 (9)
P2—C17	1.836 (6)	C24—H24	0.9500
S1—C4	1.784 (6)	C25—C26	1.414 (9)
S2—C25	1.774 (7)	C27—C28	1.528 (10)
N1—C2	1.327 (7)	C27—C29	1.556 (10)
N1—C3	1.376 (8)	C27—H27	1.0000
N2—C18	1.360 (8)	C28—H28A	0.9800
N2—C26	1.373 (7)	C28—H28B	0.9800
C1—C2	1.491 (9)	C28—H28C	0.9800
C1—H1A	0.9900	C29—H29A	0.9800
C1—H1B	0.9900	C29—H29B	0.9800
C2—C10	1.401 (9)	C29—H29C	0.9800
C3—C8	1.407 (9)	C30—C31	1.528 (9)
C3—C4	1.433 (9)	C30—C32	1.543 (9)
C4—C5	1.366 (9)	C30—H30	1.0000
C5—C6	1.405 (10)	C31—H31A	0.9800
C5—H5	0.9500	C31—H31B	0.9800
C6—C7	1.371 (10)	C31—H31C	0.9800
C6—H6	0.9500	C32—H32A	0.9800
C7—C8	1.409 (9)	C32—H32B	0.9800
C7—H7	0.9500	C32—H32C	0.9800
C8—C9	1.413 (9)	Sb1—F3	1.830 (5)
C9—C10	1.359 (9)	Sb1—F2	1.832 (5)
C9—H9	0.9500	Sb1—F6	1.845 (5)
C10—H10	0.9500	Sb1—F5	1.852 (5)
C11—C13	1.520 (10)	Sb1—F4	1.856 (4)
C11—C12	1.549 (11)	Sb1—F1	1.861 (4)
C11—H11	1.0000	Sb2—F11'	1.721 (13)
C12—H12A	0.9800	Sb2—F7	1.783 (8)
C12—H12B	0.9800	Sb2—F7'	1.784 (13)
C12—H12C	0.9800	Sb2—F8	1.796 (8)
C13—H13A	0.9800	Sb2—F12'	1.800 (13)
C13—H13B	0.9800	Sb2—F11	1.849 (7)
C13—H13C	0.9800	Sb2—F12	1.862 (6)
C14—C15	1.522 (10)	Sb2—F9	1.874 (6)
C14—C16	1.535 (10)	Sb2—F8'	1.879 (14)
C14—H14	1.0000	Sb2—F10	1.888 (8)
C15—H15A	0.9800	Sb2—F10'	1.907 (13)
C15—H15B	0.9800	Sb2—F9'	1.945 (13)
C15—H15C	0.9800	C33—Cl2	1.735 (9)
C16—H16A	0.9800	C33—Cl1	1.738 (9)
C16—H16B	0.9800	C33—H33A	0.9900
C16—H16C	0.9800	C33—H33B	0.9900
C17—C18	1.486 (9)		
			
N1—Pd1—P1	83.86 (15)	P2—C17—H17B	109.1
N1—Pd1—S2	168.93 (15)	H17A—C17—H17B	107.8
P1—Pd1—S2	106.75 (6)	N2—C18—C19	119.9 (6)
N1—Pd1—S1	86.49 (15)	N2—C18—C17	118.0 (6)
P1—Pd1—S1	169.03 (6)	C19—C18—C17	122.0 (6)
S2—Pd1—S1	82.64 (6)	C20—C19—C18	120.4 (6)
N1—Pd1—Pd2	117.54 (14)	C20—C19—H19	119.8
P1—Pd1—Pd2	129.40 (5)	C18—C19—H19	119.8
S2—Pd1—Pd2	53.28 (4)	C19—C20—C21	121.0 (6)
S1—Pd1—Pd2	51.89 (4)	C19—C20—H20	119.5
Pd2—S1—Pd1	74.71 (5)	C21—C20—H20	119.5
N2—Pd2—P2	85.21 (15)	C26—C21—C22	119.6 (6)
N2—Pd2—S1	167.55 (15)	C26—C21—C20	117.3 (6)
P2—Pd2—S1	106.34 (6)	C22—C21—C20	123.2 (6)
N2—Pd2—S2	86.13 (15)	C23—C22—C21	119.6 (7)
P2—Pd2—S2	170.20 (6)	C23—C22—H22	120.2
S1—Pd2—S2	82.69 (6)	C21—C22—H22	120.2
N2—Pd2—Pd1	114.86 (14)	C22—C23—C24	121.0 (7)
P2—Pd2—Pd1	136.83 (5)	C22—C23—H23	119.5
S1—Pd2—Pd1	53.40 (4)	C24—C23—H23	119.5
S2—Pd2—Pd1	51.83 (4)	C25—C24—C23	121.2 (7)
C11—P1—C1	106.9 (3)	C25—C24—H24	119.4
C11—P1—C14	108.9 (3)	C23—C24—H24	119.4
C1—P1—C14	105.4 (3)	C24—C25—C26	119.1 (6)
C11—P1—Pd1	119.7 (3)	C24—C25—S2	120.6 (5)
C1—P1—Pd1	98.8 (2)	C26—C25—S2	119.8 (5)
C14—P1—Pd1	115.3 (2)	N2—C26—C21	120.9 (6)
C27—P2—C30	108.7 (3)	N2—C26—C25	119.6 (6)
C27—P2—C17	107.7 (3)	C21—C26—C25	119.5 (6)
C30—P2—C17	106.1 (3)	C28—C27—C29	111.5 (6)
C27—P2—Pd2	121.4 (2)	C28—C27—P2	110.5 (5)
C30—P2—Pd2	111.0 (2)	C29—C27—P2	112.2 (5)
C17—P2—Pd2	100.6 (2)	C28—C27—H27	107.5
C4—S1—Pd2	117.9 (2)	C29—C27—H27	107.5
C4—S1—Pd1	94.8 (2)	P2—C27—H27	107.5
C25—S2—Pd1	119.8 (2)	C27—C28—H28A	109.5
C25—S2—Pd2	95.2 (2)	C27—C28—H28B	109.5
Pd1—S2—Pd2	74.88 (5)	H28A—C28—H28B	109.5
C2—N1—C3	120.8 (5)	C27—C28—H28C	109.5
C2—N1—Pd1	121.8 (4)	H28A—C28—H28C	109.5
C3—N1—Pd1	117.3 (4)	H28B—C28—H28C	109.5
C18—N2—C26	120.4 (5)	C27—C29—H29A	109.5
C18—N2—Pd2	121.4 (4)	C27—C29—H29B	109.5
C26—N2—Pd2	118.1 (4)	H29A—C29—H29B	109.5
C2—C1—P1	111.5 (4)	C27—C29—H29C	109.5
C2—C1—H1A	109.3	H29A—C29—H29C	109.5
P1—C1—H1A	109.3	H29B—C29—H29C	109.5
C2—C1—H1B	109.3	C31—C30—C32	111.5 (6)
P1—C1—H1B	109.3	C31—C30—P2	110.4 (5)
H1A—C1—H1B	108.0	C32—C30—P2	110.6 (5)
N1—C2—C10	120.9 (6)	C31—C30—H30	108.1
N1—C2—C1	117.1 (5)	C32—C30—H30	108.1
C10—C2—C1	122.0 (6)	P2—C30—H30	108.1
N1—C3—C8	120.2 (6)	C30—C31—H31A	109.5
N1—C3—C4	120.2 (6)	C30—C31—H31B	109.5
C8—C3—C4	119.6 (6)	H31A—C31—H31B	109.5
C5—C4—C3	119.2 (6)	C30—C31—H31C	109.5
C5—C4—S1	121.4 (5)	H31A—C31—H31C	109.5
C3—C4—S1	118.9 (5)	H31B—C31—H31C	109.5
C4—C5—C6	120.8 (7)	C30—C32—H32A	109.5
C4—C5—H5	119.6	C30—C32—H32B	109.5
C6—C5—H5	119.6	H32A—C32—H32B	109.5
C7—C6—C5	121.0 (7)	C30—C32—H32C	109.5
C7—C6—H6	119.5	H32A—C32—H32C	109.5
C5—C6—H6	119.5	H32B—C32—H32C	109.5
C6—C7—C8	119.7 (7)	F3—Sb1—F2	91.0 (4)
C6—C7—H7	120.1	F3—Sb1—F6	177.3 (3)
C8—C7—H7	120.1	F2—Sb1—F6	91.4 (4)
C3—C8—C7	119.6 (6)	F3—Sb1—F5	89.5 (3)
C3—C8—C9	118.1 (6)	F2—Sb1—F5	179.2 (3)
C7—C8—C9	122.3 (6)	F6—Sb1—F5	88.1 (3)
C10—C9—C8	119.8 (6)	F3—Sb1—F4	89.0 (2)
C10—C9—H9	120.1	F2—Sb1—F4	92.3 (2)
C8—C9—H9	120.1	F6—Sb1—F4	89.5 (2)
C9—C10—C2	120.2 (6)	F5—Sb1—F4	88.3 (3)
C9—C10—H10	119.9	F3—Sb1—F1	90.9 (2)
C2—C10—H10	119.9	F2—Sb1—F1	88.5 (2)
C13—C11—C12	112.5 (7)	F6—Sb1—F1	90.5 (3)
C13—C11—P1	112.6 (6)	F5—Sb1—F1	90.8 (2)
C12—C11—P1	110.8 (5)	F4—Sb1—F1	179.2 (2)
C13—C11—H11	106.8	F11'—Sb2—F7'	98.4 (10)
C12—C11—H11	106.8	F7—Sb2—F8	93.9 (6)
P1—C11—H11	106.8	F11'—Sb2—F12'	85.3 (10)
C11—C12—H12A	109.5	F7'—Sb2—F12'	95.1 (9)
C11—C12—H12B	109.5	F7—Sb2—F11	87.1 (5)
H12A—C12—H12B	109.5	F8—Sb2—F11	179.0 (5)
C11—C12—H12C	109.5	F7—Sb2—F12	93.6 (4)
H12A—C12—H12C	109.5	F8—Sb2—F12	93.7 (4)
H12B—C12—H12C	109.5	F11—Sb2—F12	85.9 (3)
C11—C13—H13A	109.5	F7—Sb2—F9	91.0 (4)
C11—C13—H13B	109.5	F8—Sb2—F9	87.7 (4)
H13A—C13—H13B	109.5	F11—Sb2—F9	92.5 (4)
C11—C13—H13C	109.5	F12—Sb2—F9	175.0 (4)
H13A—C13—H13C	109.5	F11'—Sb2—F8'	105.2 (10)
H13B—C13—H13C	109.5	F7'—Sb2—F8'	89.5 (8)
C15—C14—C16	111.0 (6)	F12'—Sb2—F8'	167.8 (10)
C15—C14—P1	110.2 (5)	F7—Sb2—F10	175.7 (5)
C16—C14—P1	112.3 (5)	F8—Sb2—F10	89.5 (5)
C15—C14—H14	107.7	F11—Sb2—F10	89.5 (5)
C16—C14—H14	107.7	F12—Sb2—F10	88.8 (4)
P1—C14—H14	107.7	F9—Sb2—F10	86.4 (4)
C14—C15—H15A	109.5	F11'—Sb2—F10'	94.0 (10)
C14—C15—H15B	109.5	F7'—Sb2—F10'	166.5 (11)
H15A—C15—H15B	109.5	F12'—Sb2—F10'	91.3 (10)
C14—C15—H15C	109.5	F8'—Sb2—F10'	82.0 (8)
H15A—C15—H15C	109.5	F11'—Sb2—F9'	171.8 (10)
H15B—C15—H15C	109.5	F7'—Sb2—F9'	89.5 (9)
C14—C16—H16A	109.5	F12'—Sb2—F9'	96.4 (9)
C14—C16—H16B	109.5	F8'—Sb2—F9'	72.3 (9)
H16A—C16—H16B	109.5	F10'—Sb2—F9'	78.0 (9)
C14—C16—H16C	109.5	Cl2—C33—Cl1	112.8 (5)
H16A—C16—H16C	109.5	Cl2—C33—H33A	109.0
H16B—C16—H16C	109.5	Cl1—C33—H33A	109.0
C18—C17—P2	112.4 (4)	Cl2—C33—H33B	109.0
C18—C17—H17A	109.1	Cl1—C33—H33B	109.0
P2—C17—H17A	109.1	H33A—C33—H33B	107.8
C18—C17—H17B	109.1		
			
C11—P1—C1—C2	−151.4 (5)	C27—P2—C17—C18	143.6 (5)
C14—P1—C1—C2	92.8 (5)	C30—P2—C17—C18	−100.2 (5)
Pd1—P1—C1—C2	−26.6 (5)	Pd2—P2—C17—C18	15.5 (5)
C3—N1—C2—C10	1.4 (9)	C26—N2—C18—C19	4.7 (9)
Pd1—N1—C2—C10	179.4 (5)	Pd2—N2—C18—C19	−176.8 (5)
C3—N1—C2—C1	−179.9 (5)	C26—N2—C18—C17	−171.1 (5)
Pd1—N1—C2—C1	−1.8 (8)	Pd2—N2—C18—C17	7.4 (8)
P1—C1—C2—N1	21.0 (7)	P2—C17—C18—N2	−15.9 (8)
P1—C1—C2—C10	−160.3 (5)	P2—C17—C18—C19	168.4 (5)
C2—N1—C3—C8	0.3 (9)	N2—C18—C19—C20	−2.4 (10)
Pd1—N1—C3—C8	−177.8 (5)	C17—C18—C19—C20	173.2 (6)
C2—N1—C3—C4	−178.9 (6)	C18—C19—C20—C21	−1.5 (10)
Pd1—N1—C3—C4	3.0 (7)	C19—C20—C21—C26	3.0 (9)
N1—C3—C4—C5	−178.2 (6)	C19—C20—C21—C22	−176.8 (7)
C8—C3—C4—C5	2.6 (9)	C26—C21—C22—C23	−1.9 (10)
N1—C3—C4—S1	10.2 (8)	C20—C21—C22—C23	177.9 (6)
C8—C3—C4—S1	−169.0 (5)	C21—C22—C23—C24	1.3 (10)
Pd2—S1—C4—C5	98.5 (5)	C22—C23—C24—C25	0.6 (11)
Pd1—S1—C4—C5	173.7 (5)	C23—C24—C25—C26	−1.8 (10)
Pd2—S1—C4—C3	−90.1 (5)	C23—C24—C25—S2	170.1 (5)
Pd1—S1—C4—C3	−14.9 (5)	Pd1—S2—C25—C24	101.8 (5)
C3—C4—C5—C6	0.0 (10)	Pd2—S2—C25—C24	177.4 (5)
S1—C4—C5—C6	171.4 (5)	Pd1—S2—C25—C26	−86.4 (5)
C4—C5—C6—C7	−2.0 (11)	Pd2—S2—C25—C26	−10.8 (5)
C5—C6—C7—C8	1.4 (11)	C18—N2—C26—C21	−3.1 (8)
N1—C3—C8—C7	177.6 (6)	Pd2—N2—C26—C21	178.3 (4)
C4—C3—C8—C7	−3.2 (10)	C18—N2—C26—C25	175.3 (5)
N1—C3—C8—C9	−1.8 (9)	Pd2—N2—C26—C25	−3.3 (7)
C4—C3—C8—C9	177.4 (6)	C22—C21—C26—N2	179.1 (6)
C6—C7—C8—C3	1.2 (11)	C20—C21—C26—N2	−0.7 (9)
C6—C7—C8—C9	−179.4 (7)	C22—C21—C26—C25	0.7 (9)
C3—C8—C9—C10	1.6 (10)	C20—C21—C26—C25	−179.1 (6)
C7—C8—C9—C10	−177.8 (7)	C24—C25—C26—N2	−177.3 (6)
C8—C9—C10—C2	0.0 (10)	S2—C25—C26—N2	10.8 (8)
N1—C2—C10—C9	−1.6 (10)	C24—C25—C26—C21	1.1 (9)
C1—C2—C10—C9	179.7 (6)	S2—C25—C26—C21	−170.8 (5)
C1—P1—C11—C13	−55.3 (7)	C30—P2—C27—C28	171.5 (5)
C14—P1—C11—C13	58.2 (7)	C17—P2—C27—C28	−74.0 (6)
Pd1—P1—C11—C13	−166.2 (5)	Pd2—P2—C27—C28	40.9 (6)
C1—P1—C11—C12	71.7 (6)	C30—P2—C27—C29	−63.5 (6)
C14—P1—C11—C12	−174.9 (5)	C17—P2—C27—C29	51.0 (6)
Pd1—P1—C11—C12	−39.2 (6)	Pd2—P2—C27—C29	165.9 (4)
C11—P1—C14—C15	−179.2 (5)	C27—P2—C30—C31	176.8 (5)
C1—P1—C14—C15	−64.7 (6)	C17—P2—C30—C31	61.2 (5)
Pd1—P1—C14—C15	43.1 (6)	Pd2—P2—C30—C31	−47.2 (5)
C11—P1—C14—C16	56.5 (6)	C27—P2—C30—C32	−59.4 (6)
C1—P1—C14—C16	170.9 (5)	C17—P2—C30—C32	−174.9 (5)
Pd1—P1—C14—C16	−81.3 (5)	Pd2—P2—C30—C32	76.7 (5)

### Hydrogen-bond geometry (Å, º)

*Cg*1 is the centroid of the C21–C26 ring.

**Table:**

*D*—H···*A*	*D*—H	H···*A*	*D*···*A*	*D*—H···*A*
C1—H1*A*···F3^i^	0.99	2.34	3.305 (9)	166
C7—H7···F1^ii^	0.95	2.37	3.229 (8)	151
C11—H11···F2^iii^	1.00	2.27	3.128 (9)	143
C17—H17*A*···F11^iv^	0.99	2.41	3.279 (10)	147
C22—H22···F8^v^	0.95	2.33	3.190 (11)	150
C23—H23···F1^iii^	0.95	2.53	3.396 (9)	152
C27—H27···F12^vi^	1.00	2.43	3.322 (10)	148
C31—H31*C*···F3^iv^	0.98	2.50	3.399 (9)	152
C33—H33*A*···F10	0.99	2.54	3.172 (13)	122
C16—H16*A*···*Cg*1	0.98	2.93	3.701 (8)	136

Symmetry codes: (i) *x*+1/2, −*y*+3/2, −*z*+1; (ii) −*x*+1, −*y*+2, −*z*+1; (iii) −*x*+1, −*y*+1, −*z*+1; (iv) *x*, −*y*+3/2, *z*+1/2; (v) −*x*+1/2, −*y*+1, *z*+1/2; (vi) −*x*+1/2, −*y*+2, *z*+1/2.

### Selected geometric parameters (Å, °)

**Table:**

Pd1-N1	2.027 (5)
Pd1-P1	2.2455 (18)
Pd1-S2	2.3149 (16)
Pd1-S1	2.3657 (17)
P1-C1	1.825 (6)
S1-C4	1.784 (6)
Pd1-Pd2	2.8425 (7)
Pd2-N2	2.027 (5)
Pd2-P2	2.2417 (18)
Pd2-S1	2.3184 (16)
Pd2-S2	2.3602 (17)
P2-C17	1.836 (6)
S2-C25	1.774 (7)
N1-Pd1-P1	83.86 (15)
N1-Pd1-S2	168.93 (15)
P1-Pd1-S2	106.75 (6)
N1-Pd1-S1	86.49 (15)
P1-Pd1-S1	169.03 (6)
S2-Pd1-S1	82.64 (6)
N1-Pd1-Pd2	117.54 (14)
P1-Pd1-Pd2	129.40 (5)
S2-Pd1-Pd2	53.28 (4)
S1-Pd1-Pd2	51.89 (4)
Pd2-S1-Pd1	74.71 (5)
Pd1-S2-Pd2	74.88 (5)
